# The laboratory parameters-derived CoLab score as an indicator of the host response in ICU COVID-19 patients decreases over time: a prospective cohort study

**DOI:** 10.1038/s41598-024-58727-y

**Published:** 2024-04-08

**Authors:** Tom Schoenmakers, Mathie P. G. Leers, Stefan H. M. Gorissen, Inge H. M. van Loo, Frank van Rosmalen, Eda Aydeniz, Joep Schellens, Marielle Driessen, Ruben Deneer, Wilhelmine P. H. G. Verboeket-van de Venne, Petra F. G. Wolffs, Walther N. K. A. van Mook, Bas C. T. van Bussel, the CoLaIC-consortium, Stephanie Ament, M. Sesmu Arbous, Otto Bekers, Miranda van Berckel, Arjan-Kars Boer, Dirk W. van Dam, Ruben Deneer, William P. T. M. van Doorn, Tom P. Dormans, Silvia M. M. A. Evers, Tim Frenzel, Madeleen de Geus-Bosma, Judith Gillis, Iwan C. C. van der Horst, W. Nadia H. Koek, Kitty Linssen, Steven J. R. Meex, Guy J. M. Mostard, Remy L. M. Mostard, Luuk C. Otterspoor, Natal A. W. van Riel, Frans Stals, Harro van Westreenen, Albert Wolthuis, Ghislaine van Mastrigt, Andrea Peeters

**Affiliations:** 1 Department of Clinical Chemistry and Hematology, Zuyderland Medical Center, Sittard-Geleen, Dr. H. Van Der Hoffplein 1, 6162 BG Sittard-Geleen, The Netherlands; 2 School of Nutrition and Translational Research in Metabolism (NUTRIM), Maastricht University, Maastricht, The Netherlands; 3 Department of Intensive Care Medicine, Maastricht University Medical Center +, Maastricht, The Netherlands; 4 Faculty of Science, Environmental Sciences, Open Universiteit, Heerlen, The Netherlands; 5 Zuyderland Academy, Zuyderland Medical Center, Sittard-Geleen/Heerlen, The Netherlands; 6 Department of Medical Microbiology, Infectious Diseases and Infection Prevention, Maastricht University Medical Center +, Maastricht, The Netherlands; 7 Care and Public Health Research Institute (CAPHRI), Maastricht University, Maastricht, The Netherlands; 8 Cardiovascular Research Institute Maastricht (CARIM), Maastricht University, Maastricht, The Netherlands; 9 School of Health Professions Education (SHE), Maastricht University, Maastricht, The Netherlands; 10 MUMC+, Maastricht, The Netherlands; 11 LUMC, Leiden, The Netherlands; 12 Radboud UMC, Nijmegen, The Netherlands; 13 Catharina Hospital, Eindhoven, The Netherlands; 14 Zuyderland MC, Sittard-Geleen/Heerlen, The Netherlands; 15 Maastricht University, Maastricht, The Netherlands; 16 Medical Center Leeuwarden, Leeuwarden, The Netherlands; 17 Technical University, Eindhoven, The Netherlands; 18 Certe, Leeuwarden, The Netherlands

**Keywords:** COVID-19, SARS-CoV-2, CoLab score, Linear mixed-effects models, Translational research, Viral infection

## Abstract

The CoLab score was developed and externally validated to rule out COVID-19 among suspected patients presenting at the emergency department. We hypothesized a within-patient decrease in the CoLab score over time in an intensive care unit (ICU) cohort. Such a decrease would create the opportunity to potentially rule out the need for isolation when the infection is overcome. Using linear mixed-effects models, data from the Maastricht Intensive Care COVID (MaastrICCht) cohort were used to investigate the association between time and the CoLab score. Models were adjusted for sex, APACHE II score, ICU mortality, and daily SOFA score. The CoLab score decreased by 0.30 points per day (95% CI − 0.33 to − 0.27), independent of sex, APACHE II, and Mortality. With increasing SOFA score over time, the CoLab score decreased more strongly (− 0.01 (95% CI − 0.01 to − 0.01) additional decrease per one-point increase in SOFA score.) The CoLab score decreased in ICU patients on mechanical ventilation for COVID-19, with a one-point reduction per three days, independent of sex, APACHE II, and ICU mortality, and somewhat stronger with increasing multi-organ failure over time. This suggests that the CoLab score would decrease below a threshold where COVID-19 can be excluded.

## Introduction

The *Severe Acute Respiratory Syndrome Corona Virus-2* (SARS-CoV-2) impacts healthcare in many different ways, such as the necessity to isolate both proven and suspected COVID-19 patients within hospital settings. Isolation requires protective efforts of healthcare personnel, such as wearing personal protective garment. During the pandemic, intensive care unit (ICU) isolation rooms were scarce, since patients with respiratory failure due to SARS-CoV-2 infection occupied such rooms for a median of 17 days^1^.

SARS-CoV-2 infected patients can be de-isolated based on clinical signs^2^, reverse transcriptase-polymerase chain reaction (RT-PCR)^3^, or rapid antigen tests^4^. The antigen test detects the n-antigen, which is active and plays an important role when the virus infects a host cell and thus can assess active viral load^5^. Nevertheless, RT-PCR testing is currently the gold standard to determine whether a patient is SARS-CoV-2 positive^6^. However, there are indications that the RT-PCR test remains positive, while infective viral particles are no longer present in the patients’ samples^7,8^.

Another method to rule out COVID-19 is the CoLab score. The CoLab score, which is based on blood parameters (see below), excludes COVID-19 when the score decreases below a certain threshold (~ − 6 for patients presenting at the emergency department). The CoLab score showed a negative predictive value of 99.5% and a sensitivity of 96.9% to rule out SARS-CoV-2 infection in COVID-19 suspected emergency department patients^9,10^. This score was subsequently clinically implemented in the emergency departments of two large Dutch teaching hospitals. In addition, the CoLab score was utilized to rule out COVID-19 in healthcare workers who presented themselves with COVID-19-related complaints^9^. Thus, the CoLab score differentiates *between* COVID-19 negative and positive patients. If the CoLab score would reflect the host response to infection and also change over time *within* COVID-19 patients, it could be a promising diagnostic and monitoring tool.

The CoLab score requires 10 chemical and hematological blood variables and the patient’s age (Table 1)^10^. Several of these blood variables reflect the activity of the immune system and therefore provide information about the actual status of the patient’s response to viral infection. As the infection progresses to the pulmonary phase, the viral replication eventually decreases and the host response takes over as the primary disease progression driver^8,11^. Due to regular blood sampling in ICU patients, the CoLab score could be a promising easy-to-use tool for monitoring the course of COVID-19 progression. Effective monitoring of the SARS-CoV-2 infection is important for de-isolation protocols.

**Table 1:** CoLab variables and coefficients.

Coefficient and variable number	Β	X	Unit
0	− 6.885		
1	0.938	Erythrocytes^a^	U/pL
2	− 0.130	Leukocytes	U/nL
3	− 6.834	Eosinophils	U/nL
4	− 47.7	Basophils	U/nL
5	− 1.142	Log_10_(Bilirubin)	µmol/L
6	5.369	Log_10_(LD)	U/L
7	− 3.114	Log_10_(ALP)	IU/L
8	0.360	Log_10_(γGT)	U/L
9	0.116	Albumin	g/L
10	0.003	CRP	mg/L
11	0.002	Age	years

^**a**^Can be inferred using haemoglobin and hAematocrit (Supplemental 1).

our hypothesis is that the CoLab score decreases over time in patients admitted to the ICU. To test this hypothesis, we need to answer several research questions:

1. does the CoLab score decrease over time within patients admitted to the ICU?
2. is this decrease affected by adjustment for mortality and acute illness? We used the Acute Physiology And Chronic Health Evaluation (APACHE) II score to investigate the effect of acute illness and pre-ICU health status. The APACHE II score has two parts: a pre-admission chronic health evaluation and an acute physiology score^12^. The first represents the health status before acute disease, and the latter reflects the severity of acute disease.
3. does multi-organ failure over time affect the association between time and the CoLab score? We used the Sequential Organ Failure Assessment (SOFA) score to investigate the effect of organ failure.

If the CoLab score decreases within patients over time, this would be the first step towards investigating the CoLab score-based early clinical SARS-CoV-2 de-isolation potential. This study is part of an overarching study design^13^.

## Materials and methods

### Patient cohort

The Maastricht Intensive Care COVID (MaastrICCht) cohort has been described elsewhere^14^. Briefly, this comprehensive prospective cohort study was conducted in patients admitted to the ICU of Maastricht University Medical Centre + (MUMC+), a tertiary care university teaching hospital in the southern part of the Netherlands^14^.

The cohort included all patients with respiratory insufficiency requiring mechanical ventilation and at least one PCR positive for SARS-CoV-2 and/or CORADS score of 4–5 scored by a radiologist (i.e., a chest CT scan strongly suggestive of SARS-CoV-2 infection)^15^. Patients were followed from intubation to the end of the ICU stay. For the present study, patients were included from March 25th, 2020, the inception of the cohort, until October 11th, 2021. ICU survival and mortality were classified either as patients who did not die during their ICU stay (survivors), or patients who died during their ICU stay (non-survivors).

### CoLab score

The CoLab score includes, in addition to a patient’s age (in years), 10 blood variables of which 9 were measured in blood samples daily drawn and analyzed according to the study protocol; leukocyte (*10^9^/L), eosinophil (*10^9^/L), basophil (*10^9^/L), bilirubin (µmol/L), lactate dehydrogenase (LD) (U/L), alkaline phosphatase (ALP) (U/L), gamma-glutamyltransferase (γGT) (U/L), albumin (g/L) and c-reactive protein (CRP) (mg/L) concentrations.

The erythrocyte concentration was not routinely measured, and the few available data on erythrocytes were deemed insufficient for imputation methods. However, other erythrocyte-related variables were measured, i.e., hemoglobin (Hb) concentration (mmol/L) and hematocrit (Hct) (L/L). We, therefore, calculated the estimated erythrocyte concentrations according to (*erythrocytes* = *0.0011 – Hb × 0.0380* + *Hct × 0.1211*) established by a generalized least squares (GLS) regression model using an external dataset (see Supplemental material chapter 1). Using the estimated erythrocyte concentrations, the other 9 laboratory variables, and age, the CoLab score was calculated daily for the entire cohort. The equation used is shown below in equation (1); the coefficient and variables are shown in Table 1.

1 $$\begin{array}{c}CoLab \; score= {\beta }_{0}+ \sum_{i=1}^{11}{\beta }_{i}\times {x}_{i} \end{array}$$

### Established ICU disease severity scores

The APACHE II score is calculated once during a patient’s admission to the ICU, whereas the SOFA score is calculated daily in the MaastrICCht cohort. The SOFA score evaluates multi-organ failure by combining six parameters that assess the respiratory system, coagulation, liver function, cardiovascular system, central nervous system, and renal function. The scores for each parameter range from 0 (indicating normalcy) to 4 (indicating the most abnormal function).

### Statistical analyses

The sample size was determined pragmatically; all eligible patients who had been enrolled in the cohort until October 11th, 2021, were included. The sample characteristics were described using mean and standard deviation (SD), median and interquartile range (IQR), or percentage, when appropriate. After analyzing missing data, the variables were imputed via multiple imputation chained equations with a decision tree algorithm (CART in the R MICE packages), as this fits regular spaced longitudinal data with low numbers of missingness in serial variables, as in this cohort^16^. The erythrocyte concentrations were inferred using the method described in Supplement 1.

Five linear mixed-effect models were used to investigate whether the CoLab score decreased over time within patients (models 1 and 2), whether this differed in survivors compared to non-survivors (model 3), and whether this was affected by multi-organ failure using the SOFA score. Concerning the latter, the model was additionally adjusted for the daily SOFA score, and then the interaction between time and SOFA score was added (models 4 and 5). The intubation day was chosen as a starting point (T_0_) for each patient, assuming that patients have the same COVID-19 severity state at intubation due to their SARS-CoV-2 infection.

In model 1, we modelled the CoLab score over time per patient with a random intercept and slope. In model 2, model 1 was adjusted for both sex and APACHE II score. The adjustment for the APACHE II score was done to adjust for disease severity at ICU admission. In model 3, model 2 was additionally adjusted for ICU mortality and also an interaction term with time was investigated to model whether any decrease in CoLab score over time was steeper in survivors than non-survivors. In model 4, we adjusted model 2 for the SOFA score to investigate whether the association between time and CoLab score was independent of multi-organ failure during ICU admission. In addition, in model 5 the SOFA score × time was added as an interaction term to investigate whether the CoLab score decreased over time (if so) even with increasing multi-organ failure, to investigate whether the CoLab score measures another aspect of the host-response than multi-organ failure^17,18^. The mixed model analyses were done in R (version: 3.1.1) using the package LME4 (version: 4.1.2). Effect estimates β and 95% confidence interval (95% CI) were reported with p-values. A two-sided p-value < 0.05 and interaction p-value < 0.10 were considered statistically significant.

### Ethics

Informed consent was obtained from all the study participants. The institutional review board (Medisch Ethische Toetsingscommissie (METC) 2020-1565/300523) of the Maastricht UMC + approved the study, which was performed following the Declaration of Helsinki. During the pandemic, the board of directors of Maastricht UMC + adopted a policy to inform patients and ask their consent to use the collected data and to store left-over serum samples for COVID-19 research purposes. The study was registered in the International Clinical Trials Registry Platform (registration number NL8613, 12/05/2020).

### Patients and public involvement

Patients (e.g., Longfonds) were and will be involved in the design and dissemination plans of this research.

## Results

### Patient cohort

**Figure 1:**
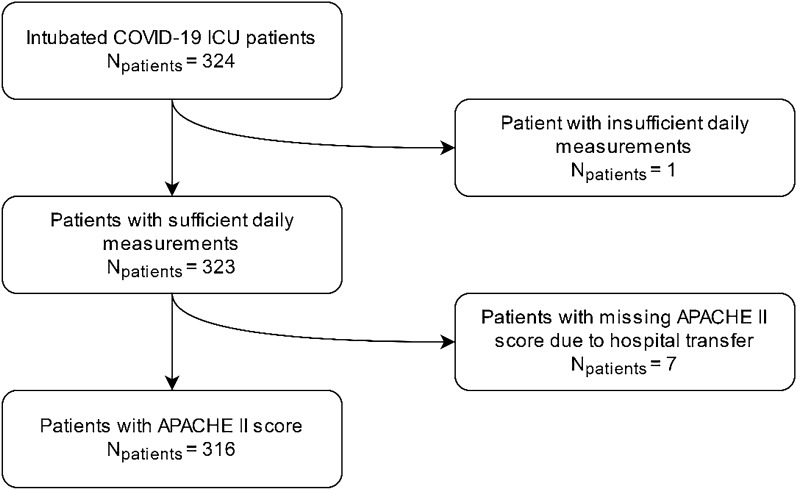
Inclusion flow chart. *ICU* intensive care unit, *APACHE* Acute Physiology and Chronic Health Evaluation.

**Figure 2:**
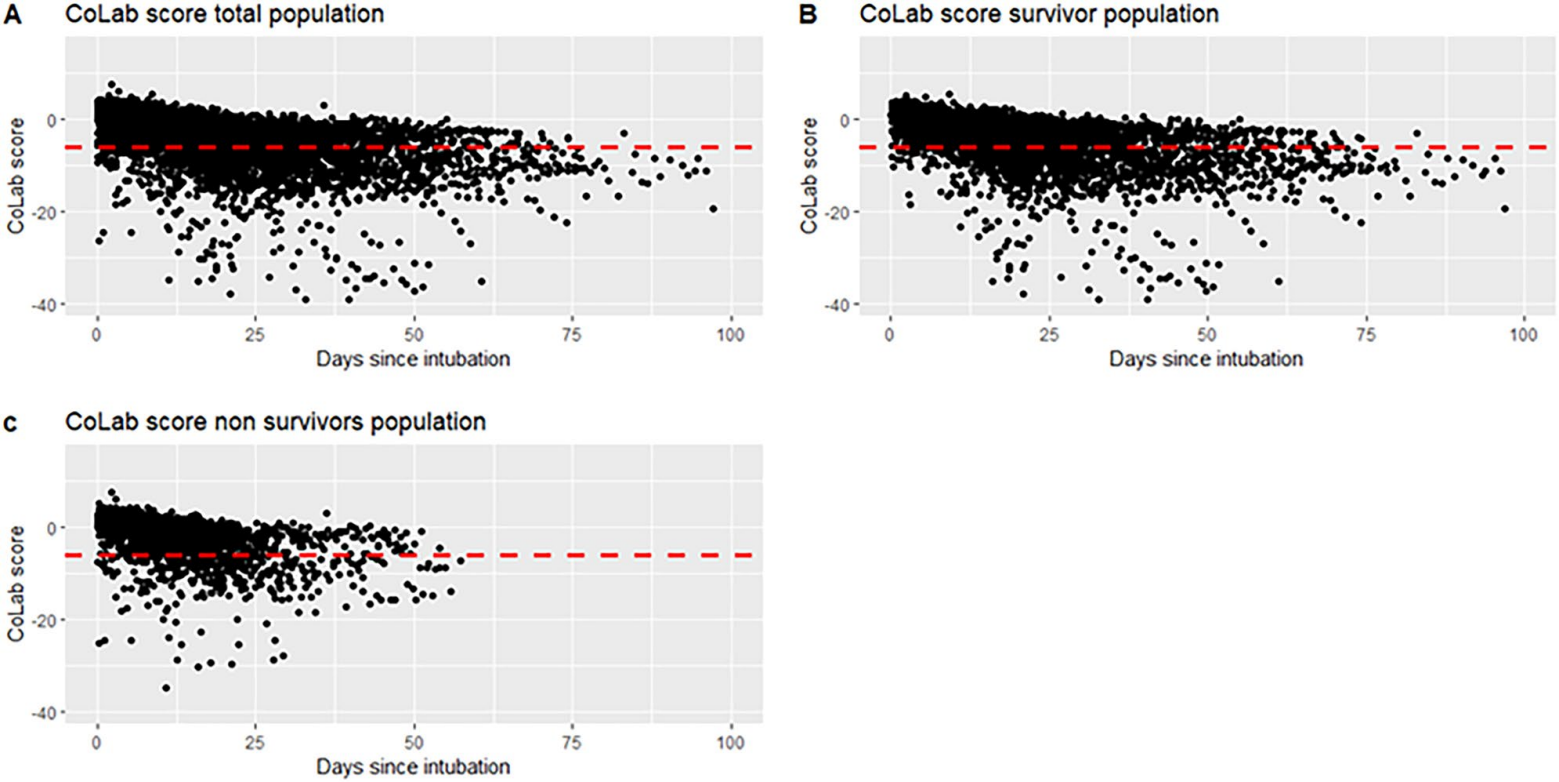
Scatter plot of the CoLab score. The CoLab score was measured daily from the start of intubation. A general downward trend can be observed. The red dotted line is the CoLab score threshold that the emergency department uses for excluding active SARS-CoV-2 infection (COVID-19 is highly unlikely below the red dotted line for the threshold that the emergency department uses)^10^. (**A**) The CoLab score over time of the total population. (**B**) The CoLab score over time of the survivor population. (**C**) The CoLab score over time of the non-survivor population.

Within the MaastrICCht cohort, 324 mechanically ventilated patients were included. 6.7% of the investigated variables were missing. For one patient insufficient daily measurements were available, leaving 323 patients for analysis (Table 2, Fig. 1). Of the patients included, 238 (73%) were male. The median age was 64 years (interquartile range 57–72) with a median length of stay (after intubation) in the ICU of 14 days (interquartile range 8–23). The median APACHE II and SOFA scores were 15 (interquartile range 12–18) and 14 (interquartile range 13–15), respectively. During the ICU stay, 126 patients died. The median CoLab score at admission was 1.3 (interquartile range 0.0–2.2). For the survivor group, the CoLab score was 1.2 (interquartile range 0.1–2.1) at admission, which was comparable to the non-survivor group 1.4 (interquartile range − 0.1 to 2.3) (Table 2). The global trend of the CoLab score was a decrease over time (Fig. 2).

**Table 2:** Characteristics of the study population; overall and stratified for survivors and non-survivors.

		Overall			Survivors			Non-survivors		
Cohort characteristics at intubation	Number of patients (n)	323			197			126		
	Number of observations (n)	5957			1996			3961		
	Sex (male (%))	237 (73.4)			137 (69.5)			100 (79.4)		
	Immunosuppression(n (%))	24 ( 7.4)			8 ( 4.1)			16 (12.7)		
		Median	IQR 25	IQR 75	Median	IQR 25	IQR 75	Median	IQR 25	IQR 75
	Age (years)	64.0	57.0	72.0	62.0	54.0	69.0	67.5	61.0	73.0
	Height (cm)	175.0	168.0	181.0	175.0	168.0	182.0	175.0	169.0	180.0
	Weight (kg)	87.0	80.0	97.0	88.0	80.0	100.0	85.0	77.0	94.8
	BMI (kg/m2)	27.8	25.4	31.4	28.2	25.9	32.3	27.6	24.7	30.8
	APACHE II	15.0	12.0	18.0	14.0	11.0	17.0	16.0	14.0	19.0
	ICU length of stay (days)	14.0	8.0	22.5	13.0	7.0	27.0	14.0	8.0	21.0
Serial variables on admission	CoLab score	1.3	0.0	2.2	1.2	0.1	2.1	1.4	-0.1	2.3
	Erythrocytes (calculated)	4.2	3.8	4.5	4.2	3.9	4.6	4.2	3.6	4.5
	Leukocytes	9.8	7.3	13.0	9.4	7.2	12.4	10.7	7.4	13.8
	Eosinophils	0.0	0.0	0.1	0.0	0.0	0.1	0.0	0.0	0.1
	Basophils	0.0	0.0	0.0	0.0	0.0	0.0	0.0	0.0	0.0
	Bilirubin	7.4	4.7	12.7	6.9	4.6	11.8	8.3	4.9	14.6
	LD	417.0	350.5	534.5	412.0	341.0	509.0	432.0	359.8	569.0
	ALP	76.0	59.0	98.5	73.0	58.0	98.0	79.0	61.8	102.8
	γ-GT	70.0	48.0	136.0	72.0	45.0	139.0	70.0	49.0	118.5
	Albumin	21.5	18.7	23.9	21.5	18.7	23.9	21.2	18.6	23.9
	CRP	126.0	48.5	233.5	113.0	47.0	214.0	157.0	63.8	261.0
	Hb	7.5	6.6	8.2	7.6	6.8	8.2	7.5	6.4	8.2
	Hct	37.0	33.0	40.0	37.0	34.0	40.0	37.0	32.0	39.8
	SOFA score	15.0	14.0	15.00	14.0	14.0	15.0	15.0	14.0	15.0

The median and interquartile ranges are given unless stated otherwise.

### Associations between time and CoLab score

**Figure 3:**
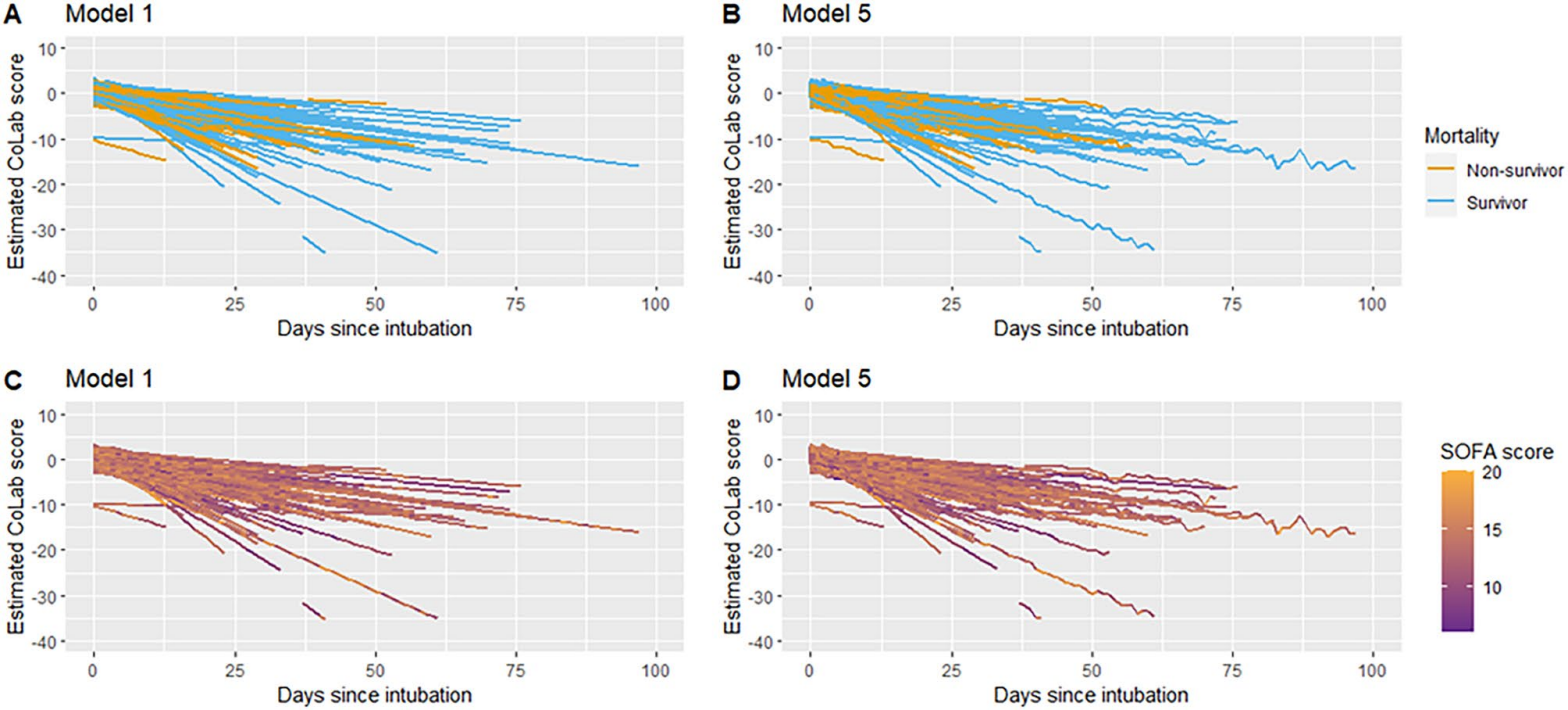
Line plot of the mixed model results. Result of the linear mixed-effects model analyses of the MaastrICCht cohort. (**A**) A blue line indicates a patient who survived the intensive care stay. An orange line indicates a patient who did not survive the intensive care stay. Results of model 1 are shown. (**B**) A blue line indicates a patient who survived the intensive care stay. An orange line indicates a patient who did not survive the intensive care stay. Results of Model 5 [adjusted for sex, APACHE II, and SOFA (predictor and interaction term)] are shown. (**C**) Results of Model 1 are shown with a heatmap related to the SOFA score (the purple color indicates a low SOFA score, while the orange color indicates a high SOFA score). (**D**) Results of Model 5 are shown with a heatmap related to the SOFA score (the purple color indicates a low SOFA score, while the orange color indicates a high SOFA score).

Model 1 showed that the CoLab score decreased by 0.30 points per day (95% CI − 0.33 to − 0.27), i.e. approximately 1 point per 3 days (Table 3). Next, adjustment for sex and APACHE II score (model 2) showed no changes in the decrease in CoLab score over time (− 0.30, 95% CI − 0.33 to − 0.27). ICU mortality adjustment did not change the decrease in CoLab score over time and the decrease did not differ between survivors and non-survivors (Interaction p-value = 0.459) (Table 3). Additional adjustment for SOFA score did not affect the association between time and CoLab score (− 0.30 95% CI − 0.33 to − 0.27). Model 5 demonstrated that patients with a higher SOFA score tended to have a higher CoLab-score (0.22 95% CI 0.12–0.31), but also showed a slightly stronger decrease over time (− 0.01 95% CI − 0.01 to − 0.01) (Table 4). The overall trend of model 5 compared with model 1 was largely the same (Fig. 3), despite some minor modifications to the intercept and the slope. The addition of the SOFA interaction over time in model 5 only appeared to model minor fluctuations of the CoLab score in time.

**Table 3:** Associations between time and CoLab score.

Variables	Model 1			Model 2			Model 3		
	Beta	CI	p	Beta	CI	p	Beta	CI	p
Intercept	0.93	0.63 to 1.22	**< 0.001**	1.80	0.78 to 2.82	**0.001**	1.74	0.44 to 3.03	**0.009**
Days since intubation	− 0.30	− 0.33 to − 0.27	**< 0.001**	− 0.30	− 0.33 to − 0.27	**< 0.001**	− 0.34	− 0.43 to − 0.24	**< 0.001**
Sex				− 0.18	− 0.74 to 0.38	0.521	− 0.16	− 0.72 to 0.41	0.588
APACHE II				− 0.04	− 0.09 to 0.01	0.079	− 0.05	− 0.09 to 0.00	0.065
ICU mortality							0.05	− 0.58 to 0.69	0.865
Days since intubation^a^ ICU mortality							0.02	− 0.04 to 0.09	0.459
Patients (N =)	323			316^a^			316^a^		
Observations	5957			5841			5841		

Significant values are in bold.

^a^For 7 patiënts the APACHE II was missing.

**Table 4:** Associations between time and CoLab score and multi-organ failure.

Variables	Model 4			Model 5		
	Beta	CI	p	Beta	CI	p
Intercept	0.96	− 0.43 to 2.35	0.176	− 1.28	− 2.97 to 0.41	0.139
Days since intubation	− 0.30	− 0.33 to − 0.27	**< 0.001**	− 0.16	− 0.23 to − 0.10	**< 0.001**
Sex	− 0.17	− 0.73 to 0.38	0.542	− 0.16	− 0.72 to 0.40	0.574
APACHE II	− 0.04	− 0.09 to 0.00	0.076	− 0.05	− 0.09 to 0.00	0.064
SOFA score	0.06	− 0.01 to 0.12	0.082	0.22	0.12 to 0.31	**< 0.001**
Days since intubation × SOFA score				− 0.01	− 0.01 to − 0.01	**< 0.001**
Patients (N =)	316^a^			316^a^		
Observations	5841			5841		

Significant values are in bold.

^a^For 7 patiënts the APACHE II was missing.

## Discussion

The main finding of this study is a decrease in the CoLab score of 0.30 points per day or about 1 point per 3 days. This observation is independent of sex and APACHE II score. This suggests that, if a sufficiently long follow-up period would be available for each patient, the CoLab score would decrease beneath a SARS-CoV-2 infection exclusion threshold in all patients.

Furthermore, the CoLab score decreased comparably in patients who survived and in patients who died during ICU stay. This suggests that the CoLab score has no prognostic value for mortality, at least in this ICU cohort, and could be investigated similarly in survivors and non-survivors for future diagnostic monitoring. Finally, the decrease in CoLab score was independent of multi-organ failure, indicated by the SOFA score as suggested by model 4. However, when taking into account the interaction with time, a higher SOFA score was associated with a higher CoLab score with a steeper decrease in CoLab score over time, meaning that when the SOFA score increases over time the CoLab score decreases more strongly. This suggests that the CoLab score, reflecting a part of the host response to infection, is independent from multi-organ failure. At the start of the study, it was unknown whether the CoLab score would decrease within patients with proven COVID-19 on mechanical ventilation in the ICU, as the CoLab score was primarily developed and externally validated to rule out a SARS-CoV-2 infection in patients with suspected SARS-CoV-2 infection in an emergency department setting^10^. In that setting, the score was determined only once, at presentation^9,10^. The present study is unique in the assessment of the development of the CoLab score over time. Longitudinal analyses showed that the CoLab score decreased after the intubation in mechanically ventilated patients.

Importantly, the CoLab score decreased regardless of ICU mortality. No statistically significant difference between survivors and non-survivors was observed concerning the CoLab score. This suggests there is no statistical evidence that the CoLab score has a prognostic value for predicting mortality. This is desirable as our research is the first step in assessing the de-isolation capabilities of the CoLab score, in both survivors and non-survivors.

Another important finding is the fact that the CoLab score decreased independently of the SOFA score (model 4). An increase in SOFA score is associated with a higher likelihood of mortality in ICU patients in general^19^ as well in patients with COVID-19^17^. More in-depth analyses using interaction terms showed that the CoLab score decreased to a greater extent with increasing SOFA score (model 5). Thus, a stronger decrease in CoLab score in patients with increased organ failure was suggested, although visual examination of the decreasing trend in the CoLab score of the model including the SOFA score (model 5) was comparable to the crude model (model 1). Nevertheless, the CoLab score decreased over time, independent of multi-organ dysfunction. This observation favours the hypothesis that the CoLab score reflects another aspect of the host response than the SOFA score.

In the original paper by Boer et al., a low CoLab score between patients (below − 5.95 in the emergency department) is associated with the likelihood of a SARS-CoV-2 infection of 0.01%^10^. The present study adds that the CoLab score decreases over time within patients. However, as our study was not designed as a diagnostic prediction model study, we did not determine an actual threshold to exclude COVID-19 for this group of patients yet. Nevertheless, our results suggest that the CoLab score will eventually decrease below this threshold, and we assume that this reflects a host response that has overcome active viral infection. Based on de present investigation the clinical utility of the score is uncertain. nonetheless, the present results cannot exclude that decreases in CoLab score over time can help to de-isolate patients better in the future. Further steps that investigate whether it is possible to rule out the need for isolation based on the CoLab score are warranted.

The objective of this study was to assess the association between time and CoLab score to assess the CoLab score as a potential tool for future monitoring and diagnostics for de-isolation, and is not intended to be an etiological investigation into causal inference^20,21^.

One of the limitations of this study is that only intubated patients from a single center were analyzed. This limits the generalizability of our results to other populations. Nevertheless, timely de-isolation of mechanically ventilated patients would reduce the use of scarce resources such as fully equipped ICU isolation rooms. the second limitation of the CoLab score calculation in our cohort was the fact that erythrocytes were sparsely measured. Hemoglobin and hematocrit were therefore used to infer the erythrocyte concentrations using linear regression analysis (Supplemental 1). The third limitation of the present study is that the effect of different variants of concern (VOC) on the temporal trends could not be assessed. This study investigated critically ill ICU patients. However, the time period did not include Omicron, a VOC. Omicron has been associated with a higher level of transmissibility and immune evasion and reduced pathogenicity compared to previous VOC in the non-hospital general population^22^. The effect of the Omicron variant on the CoLab score will consequently be investigated in a planned follow-up study as outlined by the pre-specified study design^13^.

An important strength of the present study is that data completeness was high (only 6.7% of the time series variables were missing). To prevent potential bias based on complete case analyses, we use multiple imputation. Together this contributes to a low chance of information bias in the reported associations. Furthermore, daily prospective measurements of patients’ vital signs, blood parameters, and clinical scores (e.g., SOFA score) enabled addressing the hypothesis comprehensively. Finally, the analysis using linear-mixed effect models accounted for the dependency within the data in this longitudinal dataset.

This study is the first step in assessing if the CoLab score could be used to de-isolate COVID-19 patients. The subsequent step will be to determine the COVID-19 exclusion threshold. This will be achieved by comparing the CoLab score over time with serially collected PCR samples and with validated viral culture surrogates (serially collected viability PCR^13,23^). With this analysis, we aim to demonstrate that no viable viral particles are present under a certain CoLab threshold. Then it can be assessed whether this CoLab threshold can be used to de-isolate COVID-19 patients. There is another laboratory test available to measure active viral particles called the antigen test. This test detects the N-antigen which is active and plays an important role in infecting a host cell^5^. It has a higher detection limit when compared to RT-PCR^24^. According to a systemic review by Mathur et al.^25^, the N-antigen tests generally have high specificity for infectious viral shedding, despite having a wide range of sensitivity^25^. A further aspect that needs exploration, is whether the decrease in CoLab score is also present in a less strictly defined population for reasons of generalizability. In follow-up studies, the CoLab score development over time should thus be investigated in a larger ICU population of COVID-19 patients with ICU data from different hospitals as well as in non-ICU patients in the pulmonology ward in one of these hospitals^13^.

When comparing the CoLab score to RT-PCR and antigen testing for de-isolation of SARS-CoV-2 infected patients, the use of the CoLab score has several advantages. The CoLab score utilizes frequently determined blood variables that are already used for other monitoring purposes. Using these variables, the CoLab score could be automatically calculated, enabling frequent assessment of the infectivity status of the SARS-CoV-2 infection. An additional benefit of re-using frequently tested blood variables, when compared to additional tests for infectivity, is that such a score could reduce costs as automated calculation in the electronic health record is possible for decision support. In addition, serial monitoring over time using such score is also possible, depending on the frequency of the testing of the underlying blood parameters. However, whether the CoLab score can replace or augment PCR and antigen testing for de-isolation purposes, remains unclear and obviously requires further study.

In conclusion, the CoLab score in mechanically ventilated ICU-admitted COVID-19 patients decreased over time after intubation to ICU discharge by approximately one point every 3 days. This observation was not affected by age, sex, disease severity at admission, mortality, and development of multi-organ failure. Thereby, this study provides evidence that a decrease in CoLab scores within patients can be observed. This first observation suggests that the CoLab score could eventually pass a certain threshold, possibly reflecting a host response that has overcome the SARS-CoV-2 infection. The establishment of a clear link between the CoLab score and the presence or absence of active viral particles needs to be investigated next. The findings of these additional studies aim to contribute to the development of future CoLab score-assisted de-isolation decision support.

### Supplementary Information

Supplementary Information.

## Data Availability

The datasets generated during and/or analyzed during the current study are available from the corresponding author on reasonable request.
