# Supplementary material for: The laboratory parameters-derived CoLab score as an indicator of the host response in ICU COVID-19 patients decreases over time: a prospective cohort study
Source: Sci Rep. 2024 Apr 8;14:8220. doi: 10.1038/s41598-024-58727-y (PMC11001996; doi:10.1038/s41598-024-58727-y)
Supplement: Supplementary file 1 — Supplementary Information. [file 41598_2024_58727_MOESM1_ESM.docx]

# Supplemental 1: Erythrocyte imputation

We calculated estimated erythrocyte concentrations according to supplement equation 1 established by generalized least squares (GLS) regression model using hematocrit (Hct (L/L)) and haemoglobin (Hb (mmol/L)). This GLS model was developed on cross-sectional data from 559 COVID-19 patients and internally validated with data from 138 COVID-19 patients at the emergency department in the period of March 1^st^, 2020 till May 15^th^, 2020, using the same COVID-19 criteria as the MaastrICCht cohort. Next, the model was externally validated for ICU patients using a separate ICU cohort consisting of 59 COVID-19 patients included in the period of March 29^th^, 2020 to February 10^th^, 2021.

The root-mean-square error (RMSE) and mean absolute error (MAE) of the GLS on the training dataset were 0.283 and 0.230 respectively with an r^2^ of 0.819. The optimal formula for the GLS is shown in supplement equation 1 On the emergency department validation dataset, the RMSE was 0.295 and the MAE was 0.213 with an r^2^ of 0.843. Finally, the model was validated using the ICU validation dataset where the RSME was 0.324 and the MAE was 0.239 with an r^2^ of 0.857 (Supplemental Figure 1).

$$\begin{aligned} Erythrocyte =0.0011-Hb*0.0380+Hct*0.1211 \#\left( supplement eq1 \right) \end{aligned}$$

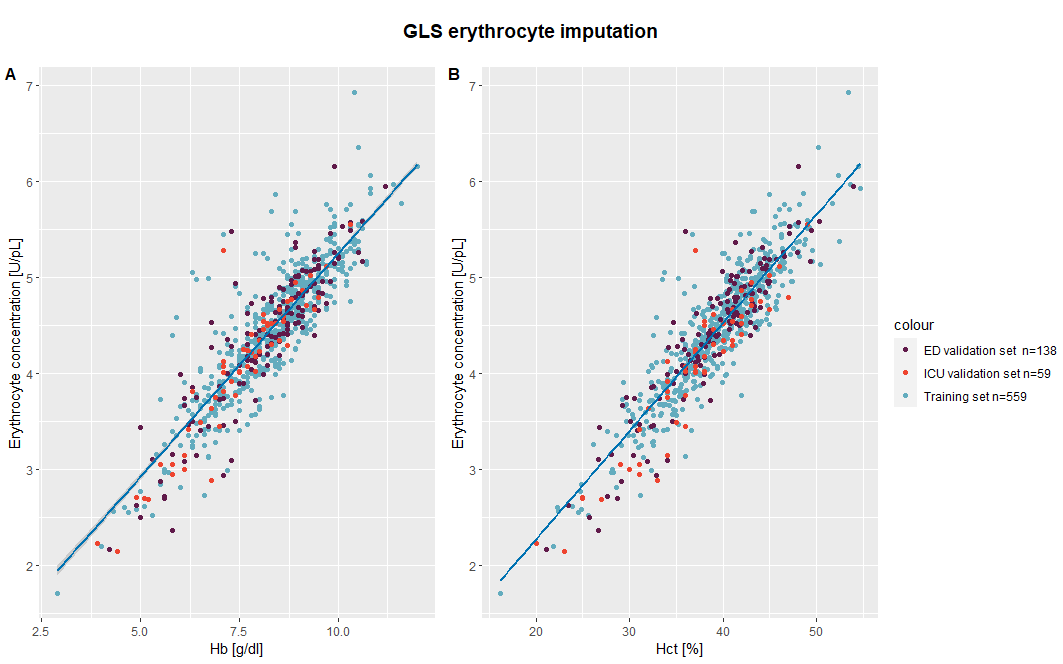


***Supplemental Figure 1: Erythrocyte imputation regression model results***

The results of the erythrocyte imputation are plotted, using Hb (A) or Hct (B). The colored dots represent the training data (blue), the emergency department validation data (purple), and the intensive care validation data (red). The line is the erythrocyte regression line.

# Supplement 2: serial variables distributions

The distributions per day are visualized to give an additional overview of the data presented in supplementary table 2.
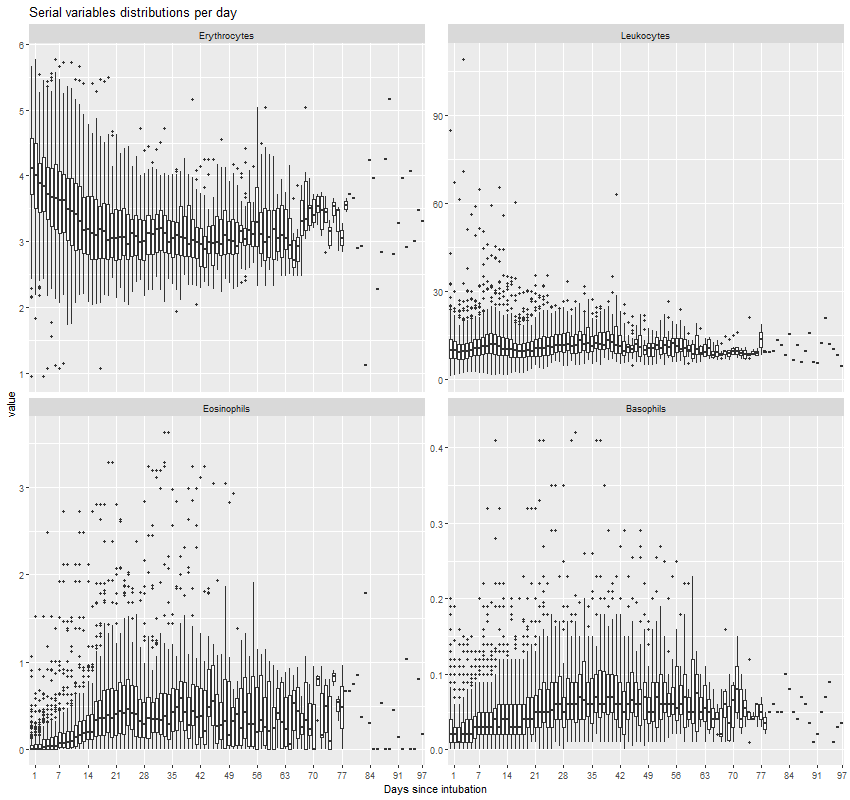


**Supplemental Figure 2: distribution of the erythrocytes, leukocytes, eosinophils and basophils**

These four figures show the distribution (boxplots) per day for the serial variables erythrocytes, leukocytes, eosinophils and basophils. Note that for the visualization different y-axis scales are used.


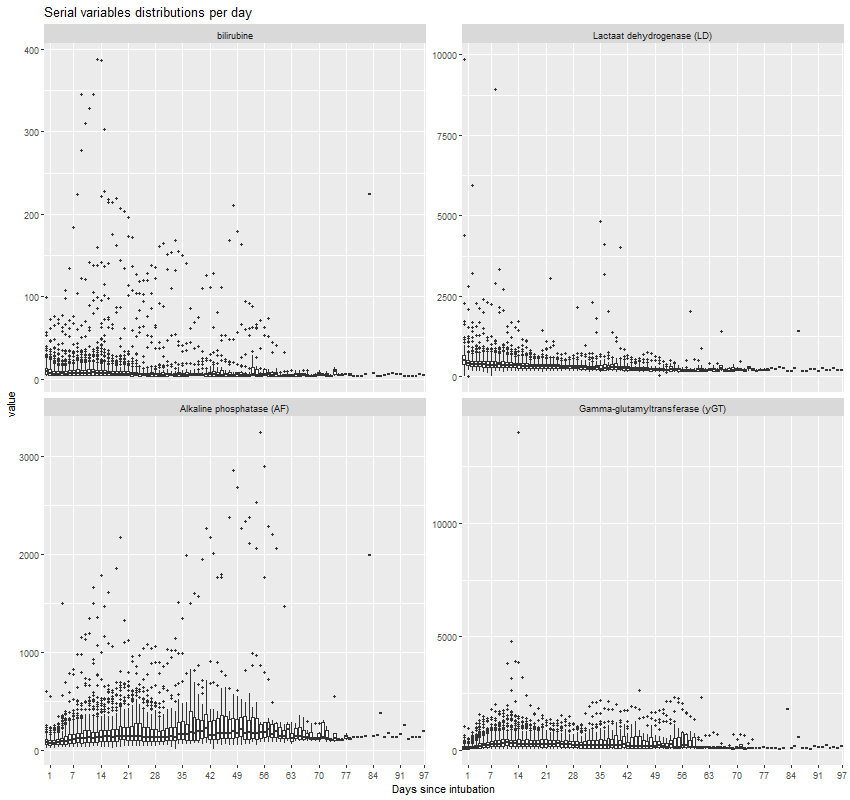


***Supplemental Figure 3: distribution of the bilirubin, Lactate dehydrogenase (LD), Alkaline phosphatase and Gamma-glutamyl transferase (yGT)***

These four figures show the distribution (boxplots) per day for the serial variables bilirubin, Lactate dehydrogenase (LD), Alkaline phosphatase and Gamma-glutamyl transferase (yGT). Note that for the visualization different y-axis scales are used.


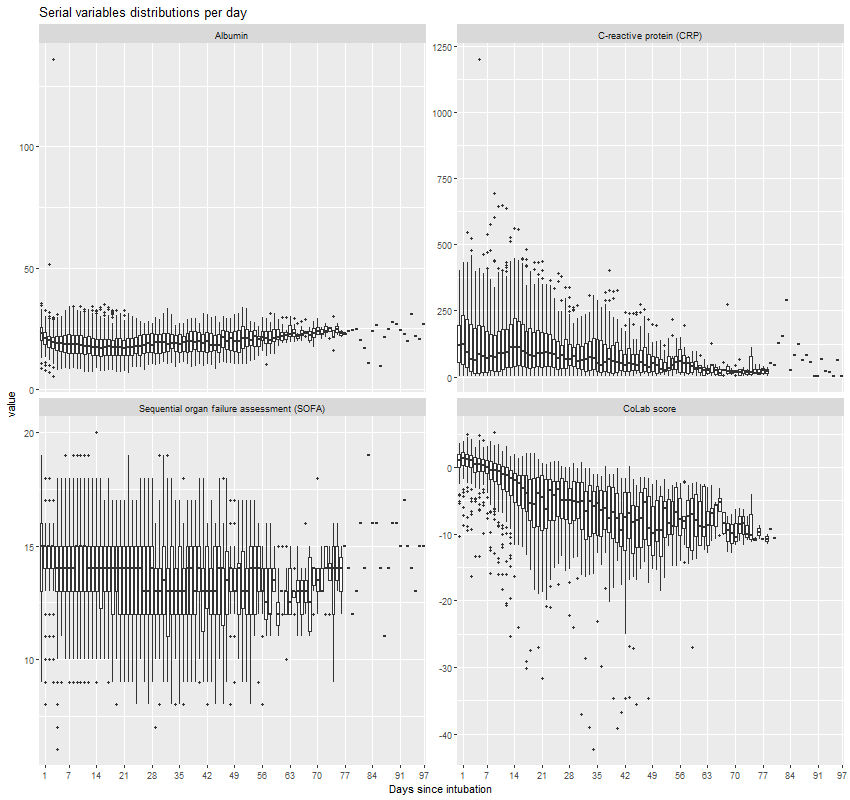


***Supplemental Figure 4: distribution of the Albumin, C-reactive protein (CRP), sequential organ failure assessment (SOFA) and CoLab score***

These four figures show the distribution (boxplots) per day for the serial variables Albumin, C-reactive protein (CRP), sequential organ failure assessment (SOFA) and CoLab score. Note that for the visualization different y-axis scales are used.


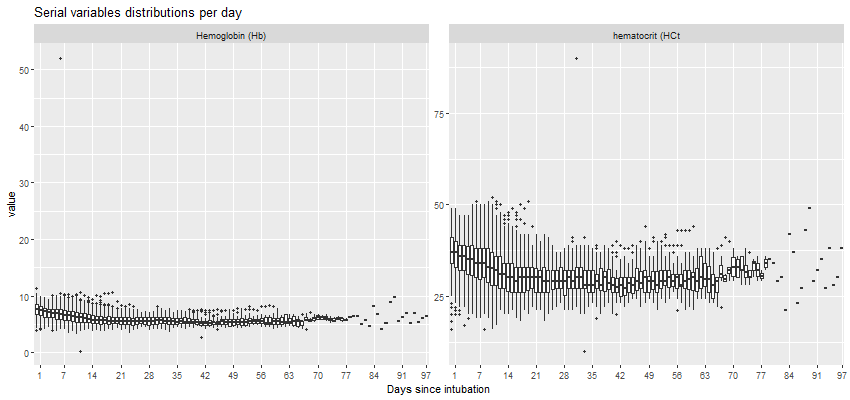


***Supplemental Figure 5: distribution of the Hemoglobin (Hb), Hematocrit (Hct)***

These four figures show the distribution (boxplots) per day for the serial variables of the Hemoglobin (Hb), Hematocrit (Hct). Note that for the visualization different y-axis scales are used.

# Supplement 3: Strobe statement

STROBE Statement—checklist of items that should be included in reports of observational studies

|  | **Item No.** | **Recommendation** | **Page  No.** |
| --- | --- | --- | --- |
| **Title and abstract** | 1 | (*a*) Indicate the study’s design with a commonly used term in the title or the abstract | 1 |
|  |  | (*b*) Provide in the abstract an informative and balanced summary of what was done and what was found | 2 |
| **Introduction** | | | |
| Background/rationale | 2 | Explain the scientific background and rationale for the investigation being reported | 3-4 |
| Objectives | 3 | State specific objectives, including any prespecified hypotheses | 3 |
| **Methods** | | | |
| Study design | 4 | Present key elements of study design early in the paper | 5 |
| Setting | 5 | Describe the setting, locations, and relevant dates, including periods of recruitment, exposure, follow-up, and data collection | 5 |
| Participants | 6 | (*a*) *Cohort study*—Give the eligibility criteria, and the sources and methods of selection of participants. Describe methods of follow-up  *Case-control study*—Give the eligibility criteria, and the sources and methods of case ascertainment and control selection. Give the rationale for the choice of cases and controls  *Cross-sectional study*—Give the eligibility criteria, and the sources and methods of selection of participants | 5 |
|  |  | (*b*) *Cohort study*—For matched studies, give matching criteria and number of exposed and unexposed  *Case-control study*—For matched studies, give matching criteria and the number of controls per case | na |
| Variables | 7 | Clearly define all outcomes, exposures, predictors, potential confounders, and effect modifiers. Give diagnostic criteria, if applicable | 5-6 |
| Data sources/ measurement | 8* | For each variable of interest, give sources of data and details of methods of assessment (measurement). Describe comparability of assessment methods if there is more than one group | *5* |
| Bias | 9 | Describe any efforts to address potential sources of bias | 6 |
| Study size | 10 | Explain how the study size was arrived at | 6 |

Continued on next page

| Quantitative variables | 11 | Explain how quantitative variables were handled in the analyses. If applicable, describe which groupings were chosen and why | | 5-6 |
| --- | --- | --- | --- | --- |
| Statistical methods | 12 | (*a*) Describe all statistical methods, including those used to control for confounding | | 6 |
|  |  | (*b*) Describe any methods used to examine subgroups and interactions | | 6 |
|  |  | (*c*) Explain how missing data were addressed | | 6 |
|  |  | (*d*) *Cohort study*—If applicable, explain how loss to follow-up was addressed  *Case-control study*—If applicable, explain how matching of cases and controls was addressed  *Cross-sectional study*—If applicable, describe analytical methods taking account of sampling strategy | | NA |
|  |  | (*e*) Describe any sensitivity analyses | | NA |
| **Results** | | |  |  |
| Participants | 13* | (a) Report numbers of individuals at each stage of study—eg numbers potentially eligible, examined for eligibility, confirmed eligible, included in the study, completing follow-up, and analysed | | 7 |
|  |  | (b) Give reasons for non-participation at each stage | | 7 |
|  |  | (c) Consider use of a flow diagram | | 15 |
| Descriptive data | 14* | (a) Give characteristics of study participants (eg demographic, clinical, social) and information on exposures and potential confounders | | 7 |
|  |  | (b) Indicate number of participants with missing data for each variable of interest | | 7 |
|  |  | (c) *Cohort study*—Summarise follow-up time (eg, average and total amount) | | NA |
| Outcome data | 15* | *Cohort study*—Report numbers of outcome events or summary measures over time | | *7* |
|  |  | *Case-control study—*Report numbers in each exposure category, or summary measures of exposure | |  |
|  |  | *Cross-sectional study—*Report numbers of outcome events or summary measures | |  |
| Main results | 16 | (*a*) Give unadjusted estimates and, if applicable, confounder-adjusted estimates and their precision (eg, 95% confidence interval). Make clear which confounders were adjusted for and why they were included | | 7 |
|  |  | (*b*) Report category boundaries when continuous variables were categorized | | NA |
|  |  | (*c*) If relevant, consider translating estimates of relative risk into absolute risk for a meaningful time period | | NA |

Continued on next page

| Other analyses | 17 | Report other analyses done—eg analyses of subgroups and interactions, and sensitivity analyses | 7 |
| --- | --- | --- | --- |
| **Discussion** | |  |  |
| Key results | 18 | Summarise key results with reference to study objectives | 7-8 |
| Limitations | 19 | Discuss limitations of the study, taking into account sources of potential bias or imprecision. Discuss both direction and magnitude of any potential bias | 9 |
| Interpretation | 20 | Give a cautious overall interpretation of results considering objectives, limitations, multiplicity of analyses, results from similar studies, and other relevant evidence | 7-8 |
| Generalisability | 21 | Discuss the generalisability (external validity) of the study results | 9 |
| **Other information** | |  |  |
| Funding | 22 | Give the source of funding and the role of the funders for the present study and, if applicable, for the original study on which the present article is based | 11 |

*Give information separately for cases and controls in case-control studies and, if applicable, for exposed and unexposed groups in cohort and cross-sectional studies.

**Note:** An Explanation and Elaboration article discusses each checklist item and gives methodological background and published examples of transparent reporting. The STROBE checklist is best used in conjunction with this article (freely available on the Web sites of PLoS Medicine at http://www.plosmedicine.org/, Annals of Internal Medicine at http://www.annals.org/, and Epidemiology at http://www.epidem.com/). Information on the STROBE Initiative is available at www.strobe-statement.org.
